# A prospective randomized study of megestrol acetate and ibuprofen in gastrointestinal cancer patients with weight loss

**DOI:** 10.1038/sj.bjc.6690077

**Published:** 1999-02

**Authors:** D C McMillan, S J Wigmore, K C H Fearon, P O'Gorman, C E Wright, C S McArdle

**Affiliations:** University Department of Surgery, Royal Infirmary, Glasgow G31 2ER, UK; University Department of Surgery, Royal Infirmary, Edinburgh, EH3 9YW, UK

**Keywords:** prospective randomized study, gastrointestinal cancer, weight loss, megestrol acetate, ibuprofen, acute phase response

## Abstract

The use of megestrol acetate in the treatment of weight loss in gastrointestinal cancer patients has been disappointing. The aim of the present study was to compare the combination of megestrol acetate and placebo with megestrol acetate and ibuprofen in the treatment of weight loss in such patients. At baseline, 4–6 weeks and 12 weeks, patients underwent measurements of anthropometry, concentrations of albumin and C-reactive protein and assessment of appetite, performance status and quality of life using EuroQol-EQ-5D and EORTC QLQ-C30. Thirty-eight and 35 patients (median weight loss 18%) were randomized to megestrol acetate/placebo or megestrol acetate/ibuprofen, respectively, for 12 weeks. Forty-six (63%) of patients failed to complete the 12-week assessment. Of those evaluable at 12 weeks, there was a decrease in weight (median 2.8 kg) in the megestrol acetate/placebo group compared with an increase (median 2.3 kg) in the megestrol acetate/ibuprofen group (*P* < 0.001). There was also an improvement in the EuroQol-EQ-5D quality of life scores of the latter group (*P* < 0.05). The combination of megestrol acetate/ibuprofen appeared to reverse weight loss and appeared to improve quality of life in patients with advanced gastrointestinal cancer. Further trials of this novel regimen in weight-losing patients with hormone-insensitive cancers are warranted. © 1999 Cancer Research Campaign


*British Journal of Cancer* (1999) **79**(3/4), 495–500
© 1999 Cancer Research Campaign
Article no. bjoc.1998.0077

### A prospective randomized study of megestrol acetate and ibuprofen in gastrointestinal cancer patients with weight loss

**DC McMillan[1], SJ Wigmore[2], KCH Fearon[2], P O'Gorman[1], CE Wright[2] and CS McArdle[1]**

[1]University Department of Surgery, Royal Infirmary, Glasgow G31 2ER, UK; [2]University Department of Surgery, Royal Infirmary, Edinburgh EH3 9YW, UK

**Summary** The use of megestrol acetate in the treatment of weight loss in gastrointestinal cancer patients has been disappointing. The aim of the present study was to compare the combination of megestrol acetate and placebo with megestrol acetate and ibuprofen in the treatment of weight loss in such patients. At baseline, 4–6 weeks and 12 weeks, patients underwent measurements of anthropometry, concentrations of albumin and C-reactive protein and assessment of appetite, performance status and quality of life using EuroQol-EQ-5D and EORTC QLQ-C30. Thirty-eight and 35 patients (median weight loss 18%) were randomized to megestrol acetate/placebo or megestrol acetate/ibuprofen, respectively, for 12 weeks. Forty-six (63%) of patients failed to complete the 12-week assessment. Of those evaluable at 12 weeks, there was a decrease in weight (median 2.8 kg) in the megestrol acetate/placebo group compared with an increase (median 2.3 kg) in the megestrol acetate/ibuprofen group ($P < 0.001$). There was also an improvement in the EuroQol-EQ-5D quality of life scores of the latter group ($P < 0.05$). The combination of megestrol acetate/ibuprofen appeared to reverse weight loss and appeared to improve quality of life in patients with advanced gastrointestinal cancer. Further trials of this novel regimen in weight-losing patients with hormone-insensitive cancers are warranted.

**Keywords:** prospective randomized study; gastrointestinal cancer; weight loss; megestrol acetate; ibuprofen; acute phase response

Weight loss in cancer patients remains a major clinical problem because it results in loss of independence and reduces the quality and duration of life (Inagaki et al, 1974; Kern and Norton, 1988; Ovesen et al, 1993). Randomized, placebo-controlled studies of heterogeneous groups of cancer patients (mainly hormone-insensitive tumours) have demonstrated that the administration of megestrol acetate can produce improvements in weight, appetite and quality of life (Loprinzi et al, 1990; Feliu et al, 1991; Tchemedyian et al, 1992). However, in similar studies in advanced gastrointestinal cancer patients, no significant gain in weight has been documented (Schmoll et al, 1991; McMillan et al, 1994*a*).

The majority of gastrointestinal cancer patients with advanced disease have evidence of an acute phase response (McMillan et al, 1994*a*; Goransson et al, 1996), and it has been reported that the presence of such a response contributes to weight loss (Falconer et al, 1994; McMillan et al, 1994*b*; Scott et al, 1996). Recent studies have shown that concentrations of the proinflammatory cytokine interleukin 6, the acute phase protein C-reactive protein and the associated metabolic events can be moderated in such patients by the administration of the non-steroidal anti-inflammatory agent ibuprofen (McMillan et al, 1995; Preston et al, 1995; Wigmore et al, 1995). We, therefore, hypothesized that down-regulating the acute phase response using ibuprofen and stimulating the appetite using megestrol acetate might be effective in reversing or halting weight loss. The results of a small pilot study (McMillan et al, 1997) in weight-losing gastrointestinal cancer patients with an acute phase protein response were encouraging and consistent with this hypothesis because there appeared to be greater weight gain compared with our published results using megestrol acetate alone (McMillan et al, 1994*a*).

The objective of the current study was to establish in a randomized placebo-controlled double-blind trial whether the combination of megestrol acetate (480 mg day$^{-1}$) and ibuprofen (1200 mg day$^{-1}$) improved weight gain and quality of life in weight-losing gastrointestinal cancer patients compared with megestrol acetate alone.

*Received 30 December 1997*
*Revised 14 April 1998*
*Accepted 28 May 1998*

*Correspondence to:* DC McMillan, University Department of Surgery, Royal Infirmary, Glasgow G31 2ER, UK

### MATERIALS AND METHODS

Patients (in two centres) with histologically proven, locally advanced or metastatic gastrointestinal cancer, with more than 5% weight loss who were receiving supportive care only, and had a life expectancy of at least 2 months were considered eligible for inclusion in the study. No patient complained of moderate or severe dysphagia and none had an obvious functional obstruction to food intake. No patient had grossly abnormal liver function tests. Other exclusion criteria included poorly controlled hypertension, congestive heart failure or a history of veno-occlusive disease.

Baseline measurements of height, weight, mid-upper arm circumference, skinfold thicknesses, total body water, albumin and C-reactive protein were carried out. Karnofsky performance status, EuroQol and EORTC QLQ-C30 quality of life measures were also recorded.

Patients included in the study were randomized to receive megestrol acetate (160 mg three times daily)/ibuprofen (400 mg three times daily) or megestrol acetate (160 mg three times daily)/placebo (three times daily), prepared in an identical form, for 12 weeks. This dose of ibuprofen has been shown previously to down-regulate the acute phase response in gastrointestinal cancer (McMillan et al, 1995; Preston et al, 1995; Wigmore et al, 1995).

**Table 1** Baseline characteristics of weight-losing gastrointestinal cancer patients

| | Megestrol acetate and placebo ($n$ = 38) | Megestrol acetate and ibuprofen ($n$ = 35) | $P$-value |
|---|---|---|---|
| Sex (F/M) | 17/21 | 13/22 | NS |
| Age (years)[a] | 72 (50–90) | 69 (52–88) | NS |
| Body mass index[a] | 18.7 (12.0–27.7) | 20.6 (14.8–26.9) | NS |
| Weight loss (%)[a] | 18 (5–33) | 18 (5–34) | NS |
| Appetite score (scale 0–10)[a] | 3.0 (0.0–10.0) | 4.0 (0.3–9.1) | NS |
| Biceps skinfold thickness (mm)[a] | 4.4 (1.8–13.0) | 4.8 (2.0–13.0) | NS |
| Triceps skinfold thickness (mm)[a] | 7.5 (2.0–23.2) | 8.0 (3.0–15.7) | NS |
| Mid-upper arm circumference (cm)[a] | 23.7 (16.0–30.2) | 24.1 (17.5–29.1) | NS |
| Total body water (l)[a] | 28.0 (18.7–45.3) | 29.5 (21.4–37.1) | NS |
| Albumin (g $l^{-1}$)[a] | 35 (20–46) | 35 (28–50) | NS |
| C-reactive protein (mg $l^{-1}$)[a] | 29 (<6–160) | 24 (<6–253) | NS |
| Cancer site | | | |
| Colorectal ($n$) | 3 | 4 | NS |
| Oesophageal ($n$) | 2 | 2 | NS |
| Gastric ($n$) | 6 | 5 | NS |
| Pancreatic ($n$) | 25 | 24 | NS |
| Cholangiocarcinoma ($n$) | 1 | 0 | NS |

[a]Values are median (range).

The above measurements were repeated at 4–6 and 12 weeks. Patients did not have surgery, radiotherapy or chemotherapy in the 6 weeks before study or during the study period. Furthermore, no patient received corticosteroids or non-steroidal anti-inflammatory drugs other than ibuprofen during the course of the study.

The study was approved by the local hospital ethical committees and all patients were informed of the purpose and procedure of the study and all gave written informed consent.

Total body water was measured using a Xitron 4000B bio-impedance spectrum analyser (Xitron Technologies, San Diego, CA, USA) as previously described (Haman et al, 1995).

To assess appetite, the patients were asked to describe their appetite on a 10-cm linear analogue scale, ranging from poor appetite to good appetite (Raben et al, 1995).

For skinfold anthropometry, measurements of biceps and triceps skinfold thickness were undertaken using Harpenden skinfold callipers (British Indicator, West Sussex, UK) and mid-upper arm circumference using a stretch-resistant tape as described previously (Heymsfield et al, 1994). These measurements were carried out because they have been reported to correlate directly with weight change (Harries et al, 1985).

Circulating C-reactive protein and albumin concentrations were measured using standard methods (McMillan et al, 1994*c*).

Karnofsky performance status: the functional ability of patients was assessed using an 11-point numerical scale and a score was given depending on the level of independence (Mor et al, 1984).

EuroQol-EQ-5D: general health state was assessed by the patient defining which statement in each of five groups best described their state of health. Also, a linear analogue scale was marked by the patient to define their present level of health (The EuroQol group, 1990).

EORTC QLQ-C30: different aspects of quality of life were assessed using this cancer-specific 30-item questionnaire which has six functional scales (physical, role, emotional, cognitive, social, global health status) and several questions relating to range of physical symptoms (Aaronson et al, 1993). Patients marked to what extent each statement applied to them.

### Statistics

Data are presented as median and range. When appropriate, differences between megestrol acetate/placebo and megestrol acetate/ibuprofen group data were tested for statistical significance using the Mann–Whitney $U$-test and Fisher's exact test. Data from different time periods within each group were tested for statistical significance using the Freidman test, and, when appropriate, comparisons of data from different time periods were carried out using the Wilcoxon signed rank test (Minitab; Minitab, State College, PA, USA).

From our previous study of megestrol acetate alone (McMillan et al, 1994*a*), the mean weight change at 6 weeks was –1.4 kg (s.d. 2.2 kg). In contrast, in the pilot study of megestrol acetate and ibuprofen (McMillan et al, 1997), the mean weight change at 6 weeks was 2.2 kg (s.d. 2.9 kg). Based on a power of 80% to detect a mean difference in weight change of 2.5 kg, at a 5% significance level, assuming a s.d. within groups of 2.6 kg, there was a minimum requirement of 19 evaluable patients in each group at 6 weeks.

### RESULTS

Seventy-three patients were included in the study. Of these, 38 and 35 were randomized to the megesterol acetate/placebo and megesterol acetate/ibuprofen groups respectively. Thirty-two patients failed to reach the 4–6 week assessment, mainly because of disease progression requiring hospital admission, and a further 14 patients failed to reach 12 weeks.

The baseline characteristics of the patients at study entry are given in Tables 1 and 2. Overall, the median weight loss of the patients entered into the study was 18%. Moreover, the patients'

© Cancer Research Campaign 1999

**Table 2** Baseline quality of life of weight-losing gastrointestinal cancer patients

| | Megestrol acetate and placebo ($n$ = 38) Median (range) | Megestrol acetate and ibuprofen ($n$ = 35) Median (range) | *P*-value |
|---|---|---|---|
| KPS[a] | 60 (50–90) | 60 (50–90) | NS |
| EuroQol-EQ-5D | 0.630 (–0.095–1.000) | 0.689 (–0.261–1.000) | NS |
| EORTC QLQ-C30 | | | |
| Physical functioning | 50 (0–100) | 60 (0–100) | NS |
| Role functioning | 83.3 (66.7–100) | 83.3 (66.7–100) | NS |
| Emotional functioning | 66.7 (0–100) | 75.0 (25.0–100) | NS |
| Cognitive functioning | 66.7 (0–100) | 83.3 (0–100) | NS |
| Social functioning | 66.7 (0–100) | 66.7 (0–100) | NS |
| Quality of life | 33.3 (0–91.7) | 33.3 (0–83.3) | NS |
| Fatigue | 55.5 (0–100) | 50.0 (11.1–100) | NS |
| Nausea and vomiting | 33.3 (0–100) | 0 (0–100) | <0.05 |
| Pain | 33.3 (0–83.3) | 33.3 (0–83.3) | NS |
| Dyspnoea | 0 (0–100) | 0 (0–100) | NS |
| Sleep disturbance | 33.3 (0–100) | 33.3 (0–100) | NS |
| Appetite loss | 66.7 (0–100) | 66.7 (0–100) | NS |
| Constipation | 0 (0–100) | 0 (0–100) | NS |
| Diarrhoea | 0 (0–100) | 0 (0–100) | NS |
| Financial difficulty | 0 (0–100) | 0 (0–100) | NS |

[a]Karnofsky performance status.

**Table 3** Change in patient characteristics and anthropometry over initial 4–6 weeks

| | Megestrol acetate and placebo ($n$ = 19) Median (range) | Megestrol acetate and ibuprofen ($n$ = 22) Median (range) | *P*-value |
|---|---|---|---|
| Change in appetite score | 3.0 (–2.0–9)* | 2.0 (–5.0–8.9)* | NS |
| Weight gain (kg) | –1.5 (–6.0–4.5) | 1.0 (–3.7–6.5)* | <0.01 |
| Change in biceps skinfold thickness (mm) | 0 (–2.4–2.0) | 0 (–3.7–0.9) | NS |
| Change in triceps skinfold thickness (mm) | –0.1 (–5.0–2.6) | 0 (–2.8–1.7) | NS |
| Change in mid-upper arm circumference (cm) | –0.6 (–5.7–0.6)* | 0.1 (–2.5–3.1) | <0.01 |
| Change in albumin (g $l^{-1}$ | 1 (–6–6) | –1 (–6–6) | NS |

*$P$ < 0.05 compared with baseline values.

median appetite score, using the linear analogue scale, was low (between 3 and 4). Both groups were similar except for the nausea and vomiting score of the EORTC QLQ-C30 questionnaire, which was higher in the group who received megestrol acetate/placebo.

Of the 41 patients who were assessed at 4–6 weeks, there was a significant decrease in the circulating concentrations of C-reactive protein, in those patients with detectable concentrations (>5 mg $l^{-1}$), in the megestrol acetate/ibuprofen group ($n$ = 10, $P$ < 0.05), but not in the megestrol acetate/placebo group ($n$ = 13, $P$ = 0.21). Of the ten patients who had detectable C-reactive protein concentrations and were given megestrol acetate and ibuprofen, after 4–6 weeks eight patients had a decrease, one patient had no change and one patient had an increase in circulating C-reactive protein concentrations. This was associated with an increase in appetite score in eight out of ten patients.

Compared with baseline values, there was a significant increase in the linear analogue appetite scores (Table 3) in both treatment groups ($P$ < 0.05). Despite this general improvement in appetite, median weight continued to decrease (–1.5 kg) and there was a significant decrease in the mid-upper arm circumference (–0.6 cm, $P$ < 0.05) in the megestrol acetate/placebo group. In contrast, there were significant increases in the median weight (1.0 kg, $P$ < 0.05) and total body water (median 1.31, $P$ < 0.05) in those receiving megestrol acetate/ibuprofen.

There was a significant increase in weight and mid-upper arm circumference in the megestrol acetate/ibuprofen group compared with the respective values in the megesterol acetate/placebo group after 4–6 weeks treatment (Table 3, Figure 1, $P$ < 0.01). On an intention-to-treat basis, at the 4–6 week assessment 2 of the 38 patients in the megestrol acetate/placebo group gained at least 2 kg in weight

© Cancer Research Campaign 1999 

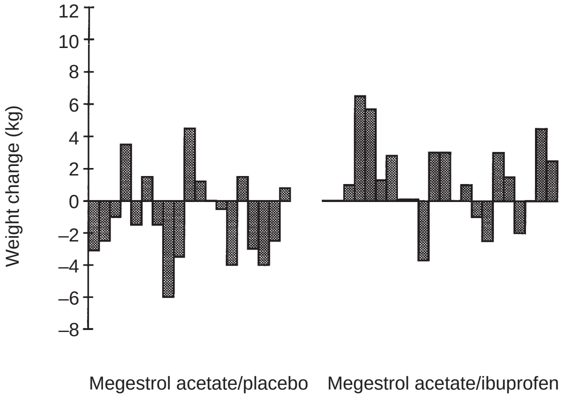

**Figure 1** Weight change after 6 weeks in gastrointestinal cancer patients randomized to receive treatment with megestrol acetate/placebo ($n$ = 19) or megestrol acetate/ibuprofen ($n$ = 22) combination

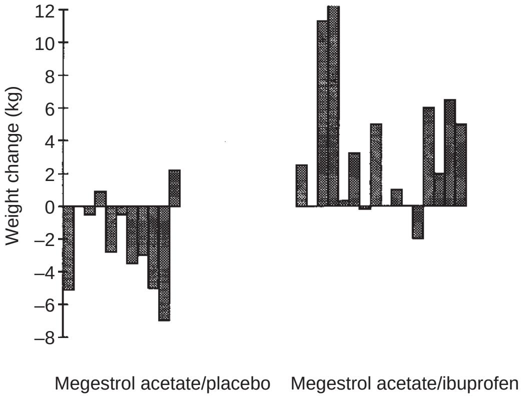

**Figure 2** Weight change after 12 weeks in gastrointestinal cancer patients randomized to receive treatment with megestrol acetate/ placebo ($n$ = 11) or megestrol acetate/ibuprofen ($n$ = 16) combination

**Table 4** Change in patient characteristics and anthropometry over 12 weeks

| | **Megestrol acetate and placebo ($n$ = 11)** | **Megestrol acetate and ibuprofen ($n$ = 16)** | ***P*-value** |
|---|---|---|---|
| | **Median (range)** | **Median (range)** | |
| Change in appetite score | 1.0 (–3.0–9.2) | 1.0 (–5.0–8.1) | NS |
| Weight gain (kg) | –2.8 (–7.0–2.2)* | 2.3 (–2.0–12.4)* | <0.001 |
| Change in biceps skinfold thickness (mm) | –0.5 (–3.3–0) | –0.2 (–4.4–3.0) | NS |
| Change in triceps skinfold thickness (mm) | 0 (–4.8–1.8) | 0.1 (–0.6–6.0)* | NS |
| Change in mid-upper arm circumference (cm) | –1.0 (–5.7–0.4)* | 0 (–5.4–3.0) | <0.05 |
| Change in albumin (g $l^{-1}$ | 0 (–8–3) | –2 (–5–1)* | NS |

*$P$ < 0.05 compared with baseline values.

compared with 8 of the 35 patients in the megestrol acetate/ibuprofen group ($P$ = 0.063). There was no significant change in total body water between the two groups.

Compared with baseline values, at 4–6 weeks there was a significant increase in the EORTC QLQ-C30 appetite scores in both treatment groups ($P$ < 0.05). However, comparing the two groups, there were no differences in any of the quality of life parameters measured.

After 12 weeks, compared with baseline values, there was a significant decrease in median body weight (–2.8 kg) in the megestrol acetate/placebo group and an increase (2.3 kg) in the megestrol acetate/ibuprofen group (Table 4, $P$ < 0.05). These changes were accompanied by a significant decrease in the mid-upper arm circumference of the megestrol acetate/placebo group ($P$ < 0.05).

After 12 weeks of treatment, there was a significant difference between the median weight gain in the megesterol acetate/ibuprofen group compared with median weight loss in the megestrol acetate/placebo group (Table 4, Figure 2, $P$ < 0.001). On an intention-to-treat basis, at the 12-week assessment only 1 of the 38 patients in the megestrol acetate/placebo group gained at least 2 kg in weight compared with 9 of the 35 patients in the megestrol acetate/ibuprofen group ($P$ < 0.01). At 12 weeks, there were insufficient total body water observations to permit meaningful statistical analysis of body composition.

Comparing the two groups at 12 weeks, there was a significant improvement in the EuroQol-EQ-5D quality of life score of the megesterol acetate/ibuprofen group ($P$ < 0.05).

During the study, venous thrombosis was recorded in three patients (one receiving megestrol acetate/placebo and two receiving megestrol acetate/ibuprofen) and upper gastrointestinal bleeding was recorded in three patients (one, fatal, receiving megestrol acetate/placebo and two receiving megestrol acetate/ibuprofen). All of these patients had locally invasive pancreatic cancer and were treated conservatively. There were clinically detectable ascites in five patients, two receiving megestrol acetate/placebo and three receiving megestrol acetate/ibuprofen. Only three patients, one in the megestrol acetate/placebo group and two in the megestrol acetate/ibuprofen group, were available for assessment at 12 weeks. No weight correction for such ascites was carried out. There was no clinically apparent congestive heart failure or deterioration in glucose tolerance observed in any of the patients studied. Failure of patients to reach assessment points (other than those detailed above) was due to disease progression resulting in changes in supportive care/pain control.

© Cancer Research Campaign 1999

### DISCUSSION

We have previously reported that the presence of an inflammatory response (as documented by an elevated C-reactive protein) is associated with increased weight loss in cancer patients (Scott et al, 1996; McMillan et al, 1997). In the present study, ibuprofen was given to attenuate the inflammatory response, mediated in part by proinflammatory cytokines such as interleukin 6 and the corticosteroids such as cortisol (McMillan et al, 1995), and as a consequence normalize host metabolism (Preston et al, 1995; Wigmore et al, 1995). The results of the present study are consistent with the anti-inflammatory effects of ibuprofen because there was a significant reduction in circulating C-reactive protein concentrations of those patients with a detectable acute phase protein response in the megestrol acetate/ibuprofen group.

In the present study, the megestrol acetate/placebo group lost weight at both 4–6 weeks (median 1.5 kg) and 12 weeks (median 2.8 kg) assessments. In contrast, the administration of megestrol acetate/ibuprofen was associated with weight gain after 4–6 weeks (median 1.0 kg) and 12 weeks (median 2.3 kg). The amount of weight lost in the megestrol acetate alone group and the amount of weight gained in the megestrol acetate/ibuprofen group was similar to our previous studies in weight-losing gastrointestinal cancer patients (McMillan et al, 1994*a*, 1997).

At 12 weeks, in the megestrol acetate/ibuprofen treatment group, circulating albumin concentrations were decreased compared with baseline values. In some circumstances, this may be consistent with fluid retention. However, such concentrations in cancer patients are dependent on a number of other factors (Fearon et al, 1998).

To examine the nature of the changes in body weight, total body water volumes were measured in the majority of patients at the 4–6 week assessment. There was a significant increase in total body water in the megestrol acetate/ibuprofen treatment group compared with baseline. This occurred in the absence of clinically detectable oedema in the majority of the patients, thereby suggesting that at least a proportion of the weight gained was lean body mass. In this study, despite measuring total body water, we were unable to determine unequivocally the nature of the weight gained.

There have been few studies which have examined the relationship between weight loss, performance status and quality of life in cancer patients. Ovesen and colleagues (1993) have reported that, in a mixed cohort of patients with lung, breast and ovarian cancer, weight loss is associated with reduced performance status and lower quality of life scores. Therefore, it is of interest that in the present study an increase in weight in the megestrol acetate/ibuprofen group was associated with an improvement in quality of life when compared with the megestrol acetate/placebo group.

Studies of palliative treatment in advanced cancer patients are fraught with difficulties in terms of analysis and interpretation of clinical relevance. Firstly, the attrition rate in such studies is high (63% at 12 weeks in the present study), and this contributes to a constantly changing patient population. Secondly, there is a need in such studies to examine changes from baseline which are clinically relevant. One approach to address these problems is to analyse the results on an intention-to-treat basis and examine the proportion of the cohort who had gained benefit. In the present study, our main finding was an increase in body weight in the megestrol acetate/ibuprofen group. Previous reports have suggested that a clinically relevant increase in weight would be approximately 2 kg (Loprinzi et al, 1990; Feliu et al, 1991; Tchemedyian et al, 1992). We have, therefore, analysed the results of the present study taking the above factors into account. At 12 weeks, significantly more patients in the megestrol acetate/ibuprofen group gained at least 2 kg in weight. This would suggest that these findings are of clinical significance. Although these results demonstrate that weight gain can be achieved by the combination of megestrol acetate plus ibuprofen, we cannot exclude the possibility that ibuprofen alone would have been as effective.

The results of the present study suggest that weight loss in gastrointestinal cancer patients can be reversed using the combination of megestrol acetate and ibuprofen. Furthermore, this weight gain may be associated with an improvement in quality of life. The nature of the weight gain and whether maintaining or increasing weight improves the survival of such cachectic patients remains to be determined. Nevertheless, this non-toxic regimen may be of therapeutic benefit in other cancers in which weight loss is associated with the presence of an inflammatory response.

### ACKNOWLEDGEMENTS

This work was supported by the Scottish Home and Health Department. The authors gratefully acknowledge the interest and encouragement of Professor TG Cooke and Professor OJ Garden.

### REFERENCES

Aaronson NK, Ahmedzai S, Bergman B, Bullinger M, Cull A, Duez NJ, Filiberti A, Flechtner H, Fleishman SB, DeHaes JCJM, Kaas S, Klee M, Obosa D, Razavi D, Rofe PB, Schraub S, Sneeuw K, Sullivan M and Takeda F (1993) The European organisation for research and treatment of cancer QLQ-C30. A quality of life instrument for the use in international clinical trials in oncology. *J Natl Cancer Inst* **85**: 365–376

Falconer JS, Fearon KCH, Plester CE, Ross JA and Carter DC (1994) Cytokines, the acute phase response, and resting energy expenditure in cachectic patients with pancreatic cancer. *Ann Surg* **219**: 325–331

Fearon KCH, Falconer JS, Slater C, McMillan DC, Ross JA and Preston T (1998) Albumin synthesis rates are not decreased in hypoalbuminaemic cachectic cancer patients with an ongoing acute phase protein response. *Ann Surg* **227**: 249–254

Feliu J, Gonzalez Baron BA, Ordonez A and Baron Saura JM (1991) Treatment of cancer anorexia with megestrol acetate: which is the optimal dose? *J Natl Cancer Inst* **83**: 449–450

Goransson J, Jonsson S and Lasson A (1996) Pre-operative plasma levels of C-reactive protein, albumin and various plasma protease inhibitors for the pre-operative assessment of operability and recurrence in cancer surgery. *Eur J Surg Oncol* **22**: 607–617

Hannan WJ, Cowen SJ, Plester CE, Fearon KCH and deBeau A (1995) Comparison of bio-impedance spectroscopy and multi-frequency bio-impedance analysis for the assessment of extracellular and total body water in surgical patients. *Clin Sci* **89**: 651–658

Harries AD, Jones LA, Heatley RHV, Newcombe RG and Rhodes J (1985) Precision of anthropometric measurements: the value of mid-arm circumference. *Clin Nutr* **4**: 77–801

Heymsfield SB, Tighe A and Wang ZM (1994) Nutritional assessment by anthropometric and biochemical methods. In *Modern Nutrition in Health and Disease*, Shils ME, Olson JA and Shike M (eds.), pp. 816–841. Lea and Febiger: Philadelphia

Inagaki J, Rodriguez V and Bodey GP (1974) Causes of death in cancer patients. *Cancer* **33**: 568–573

Kern KA and Norton JA (1988) Cancer cachexia. *Journal of Parenteral and Enteral Nutrition* **12**: 286–298

Loprinzi CL, Ellison NM, Schaid DJ, Krook JE, Athmann LM, Dose AM, Mailliard JA, Johnson PS, Ebbert LP and Geeraerts LH (1990) Controlled trial of megestrol acetate for the treatment of cancer anorexia and cachexia. *J Natl Cancer Inst* **82**: 1127–1132

McMillan DC, Simpson JM, Preston T, Watson WS, Fearon KCH, Shenkin A, Burns HJG and McArdle CS (1994*a*) Effect of megestrol acetate on weight loss, body composition and blood screen of gastrointestinal cancer patients. *Clin Nutr* **13**: 85–89

© Cancer Research Campaign 1999

McMillan DC, Preston T, Watson WS, Simpson JM, Fearon KCH, Shenkin A, Burns HJG and McArdle CS (1994*b*) The relationship between weight loss, reduction of body cell mass and the inflammatory response in cancer patients. *Br J Surg* **81**: 1011–1014

McMillan DC, Graham AF, Smith J, Wotherspoon HA, Fearon KCH and McArdle CS (1994*c*) Interleukin-6, neutrophilia and the acute phase protein response in colorectal cancer patients. *Eur J Surg Oncol* **20**: 151–154

McMillan DC, Leen E, Smith J, Sturgeon CM, Preston T, Cooke TG and McArdle CS (1995) Effect of extended ibuprofen administration on cortisol, interleukin-6 and the acute phase protein response in colorectal cancer patients. *Eur J Surg Oncol* **21**: 531–534

McMillan DC, O'Gorman P, Fearon KCH and McArdle CS (1997) A pilot study of megestrol acetate and ibuprofen in the treatment of cachexia in gastrointestinal cancer patients. *Br J Cancer* **76**: 788–790

Mor V, Laliberte L, Morris JN and Wiemann M (1984) The Karnofsky performance status scale: an examination of its reliability and validity in a research setting. *Cancer* **53**: 2002–2007

Ovesen L, Hannibal J and Mortensen EL (1993) The interrelationship of weight loss, dietary intake, and quality of life in ambulatory patients with cancer of the lung, breast and ovary. *Nutr Cancer* **19**: 159–167

Preston T, Fearon KCH, McMillan DC, Winstanley FP, Slater R, Shenkin A and Carter DC (1995) Effect of ibuprofen on the acute phase response and protein metabolism in patients with cancer and weight loss. *Br J Surg* **82**: 229–234

Raben A, Tagliabue A and Astrup A (1995) The reproducibility of subjective appetite scores. *Br J Nutr* **73**: 517–530

Schmoll E, Wilke H, Thole R, Preusser P, Wildfang I and Schmoll HJ (1991) Megestrol acetate in cancer cachexia. *Semin Oncol* **18** (suppl. 2): 32–34

Scott HR, McMillan DC, Crilly A, McArdle CS and Milroy R (1996) The relationship between weight loss and interleukin-6 in non-small cell lung cancer. *Br J Cancer* **73**: 1560–1562

Tchemedyian NS, Hickman MS J, Greco FA, Keller J, Browder H and Aisner J (1992) Megestrol acetate in cancer anorexia and weight loss. *Cancer* **69**: 1268–1274

The EuroQol Group (1990) EuroQo – a new facility for the measurement of health-related quality of life. *Health Policy* **16**: 199–208

Wigmore SJ, Falconer JS, Plester CE, Ross JA, Maingay JP, Carter DC and Fearon KCH (1995) ibuprofen reduces energy expenditure and acute phase protein production compared with placebo in pancreatic cancer patients. *Br J Cancer* **72**: 185–188

 *© Cancer Research Campaign 1999*
